# Comprehensive Study of Gene and microRNA Expression Related to Epithelial-Mesenchymal Transition in Prostate Cancer

**DOI:** 10.1371/journal.pone.0113700

**Published:** 2014-11-19

**Authors:** Betina Katz, Sabrina T. Reis, Nayara I. Viana, Denis R. Morais, Caio M. Moura, Nelson Dip, Iran A. Silva, Alexandre Iscaife, Miguel Srougi, Katia R. M. Leite

**Affiliations:** Division of Urology and Laboratory of Medical Investigation (LIM55), University of Sao Paulo Medical School, Sao Paulo, Brazil; Wayne State University School of Medicine, United States of America

## Abstract

Prostate cancer is the most common cancer in men, and most patients have localized disease at the time of diagnosis. However, 4% already present with metastatic disease. Epithelial-mesenchymal transition is a fundamental process in carcinogenesis that has been shown to be involved in prostate cancer progression. The main event in epithelial-mesenchymal transition is the repression of E-cadherin by transcription factors, but the process is also regulated by microRNAs. The aim of this study was to analyze gene and microRNA expression involved in epithelial-mesenchymal transition in localized prostate cancer and metastatic prostate cancer cell lines and correlate with clinicopathological findings. We studied 51 fresh frozen tissue samples from patients with localized prostate cancer (PCa) treated by radical prostatectomy and three metastatic prostate cancer cell lines (LNCaP, DU145, PC3). The expression of 10 genes and 18 miRNAs were assessed by real-time PCR. The patients were divided into groups according to Gleason score, pathological stage, preoperative PSA, biochemical recurrence, and risk group for correlation with clinicopathological findings. The majority of localized PCa cases showed an epithelial phenotype, with overexpression of E-cadherin and underexpression of the mesenchymal markers. MiRNA-200 family members and miRNAs 203, 205, 183, 373, and 21 were overexpressed, while miRNAs 9, 495, 29b, and 1 were underexpressed. Low-expression levels of miRNAs 200b, 30a, and 1 were significantly associated with pathological stage. Lower expression of miR-200b was also associated with a Gleason score ≥8 and shorter biochemical recurrence-free survival. Furthermore, low-expression levels of miR-30a and high-expression levels of Vimentin and Twist1 were observed in the high-risk group. Compared with the primary tumor, the metastatic cell lines showed significantly higher expression levels of miR-183 and Twist1. In summary, miRNAs 200b, 30a, 1, and 183 and the genes Twist1 and Vimentin might play important roles in the progression of prostate cancer and may eventually become important prognostic markers.

## Introduction

Prostate cancer (PCa) is one of the most common tumors in men, and it accounts for 29% of all newly diagnosed cancers [1]. After the adoption of PSA screening, most patients present with localized PCa, but 4% already have metastatic disease at the time of diagnosis [1]. At present, clinicopathological features such as staging, Gleason score (GS), and PSA levels are good prognostic markers [2] and are used to make treatment decisions; however, they are not sufficiently accurate to discriminate between tumors that will remain indolent and those that will later progress to become metastatic. Indeed, the unique biological features and heterogeneous genetic backgrounds of PCa [3] can limit the efficacy of conventional clinicopathological parameters as predictive markers. For these reasons, molecular biomarkers have been increasingly investigated to help understand and predict cancer behavior.

The epithelial-to-mesenchymal transition (EMT) is a reverse biological process that plays a role in invasion and metastasis during carcinogenesis. Epithelial cell-cell adhesion is decreased, and the cells acquire a spindle-shaped, highly motile fibroblast phenotype and a greater capacity for migration and invasion [4]. The main feature of EMT is transcriptional silencing of E-cadherin [5], [6], which is controlled by the transcriptional regulators *ZEB1*, *ZEB2*, *SNAI1* (Snail), *SNAI2* (Slug), and *TWIST1* [5], [7], [8]. Additionally, there is also upregulation of mesenchymal markers, such as Vimentin and N-cadherin, a process that is known as cadherin switching [9].

The roles of genes related to EMT in PCa are not completely understood, and previous studies describe the loss of E-cadherin [10] followed by increased expression of N-cadherin, Cadherin-11 and Vimentin [9] in immunohistochemistry analysis. The expression levels of *ZEB1*, a crucial regulator of EMT in PCa, are related to the GS [11], and Behnsawy et al proposed the use of EMT gene expression profiles as markers of biochemical recurrence after radical prostatectomy [12].

MicroRNAs (miRNAs), a new class of non-coding, regulatory RNAs, have been shown to participate in many processes related to the development and progression of cancer, including EMT [13]. One of the main miRNAs involved in EMT is the miR-200 family, which is a potent inducer of epithelial differentiation. This group comprises miR-200a, miR-200b, miR-429, miR-200c, and miR-141, which are generated from two transcripts. The first three are derived from chromosome 1, while the latter two are derived from chromosome 12. The members of this group are highly related in sequence, indicating that they likely target a similar complement of messenger RNAs [14].

Among the targets of the miR-200 family are ZEB1 and ZEB2 [15]–[17]. miR-200 members inhibit the expression of ZEB at the post-transcriptional level by binding to highly conserved target sites in their 3′UTRs [18], [19]. Interestingly, miR-200 members are transcriptional targets of *ZEB1* and *ZEB2*. The close functional link between the ZEB factors and the miR-200 family in a double-negative feedback loop is known as the ZEB/miR-200 feedback loop [18], in which the activation of one group negatively affects the expression of the other group. Depending on the extracellular signals, this loop can switch from one side to the other side and stabilize either the epithelial or mesenchymal phenotype. Other miRNAs have also been shown to participate in EMT, targeting *SNAI1* (miR-29b, miR-30a, miR-34a) [20], [21], and *SNAI2* (miR-34a, miR-1, miR-200b) [22], [23]. However, few studies have assessed miRNAs involved in EMT in PCa.

Our aim is to decipher the role of genes and miRNAs related to EMT in PCa to identify a profile that defines PCa behavior.

## Materials and Methods

### Patient selection

Fifty-one patients who had clinically localized prostate cancer and underwent radical prostatectomy between 2000 and 2002 were selected. All patients were treated by the same surgeon (MS), and all pathological specimens were analyzed by the same uropathologist (KRML). The patients were followed up for a mean time period of 63.06 months.

The control group consisted of ten samples from patients who underwent surgery for benign prostatic hyperplasia, and had prostate volume <50 cm^3^ on ultrasound, PSA levels <2,5 ng/ml, and no malignancy in the pathological specimen.

### Prostate tissue samples

All fresh-frozen PCa samples were obtained from our prostate biobank, and written informed consent was obtained from all patients. This study was approved by the institutional board of ethics (CAPPesq – Comissão de Ética para Análise de Projetos de Pesquisa) under the number 5907. The fresh-frozen tumors originated from radical prostatectomy specimens, and a 1 cm^3^ fragment was isolated from the suspicious area and immediately snap-frozen at −80°C. The remaining tissue was fixed in 10% formalin, routinely processed, and stained with hematoxylin and eosin for histological examination. The samples were subsequently reviewed and graded using the modified Gleason grading system [24], and the stage was determined following TNM 2010.

### Cell lines

The prostate cancer cell lines LNCaP, DU145, and PC3 were obtained from the American Type Culture Collection (ATCC). LNCaP, DU145, and PC3 were maintained in RPMI, DMEM, and MEM media (Invitrogen, Carlsbad, CA, EUA), respectively. All media were supplemented with 10% fetal bovine serum and a 1% antibiotic/antimycotic solution (Sigma, St. Louis, MO, USA), and the cultures were incubated at 37°C in an atmosphere of 5% CO_2_.

### RNA and miRNA isolation and amplification

Both RNA and miRNA were isolated from prostate tissues and cell lines using the Ambion mirVana kit (Austin, TX, USA) according to the manufacturer's protocol. cDNA was generated from RNA and miRNA using a TaqMan RNA Reverse Transcription Kit and TaqMan MicroRNA Reverse Transcription Kit, respectively. For gene and miRNA amplification, a TaqMan Reagent Kit was used with the 7500 Fast Real-Time PCR System (Applied Biosystems, Foster City, CA, USA). The reactions were performed in duplicate, and *B2M* (β-2-microglobulin) and RNU-48 were used as endogenous controls for genes and miRNAs, respectively.

Gene and miRNA expression levels were obtained by relative quantification using the 2^–ΔΔct^ method. The formula employed is ΔΔCT  =  dCT_1_– dCT_2_, where dCT_1_  =  CT of the target (tumor sample) – CT of the mean of the endogenous control (tumor sample), and dCT_2_  =  CT of the mean of the normal controls (benign prostate tissue) – CT of the mean of the endogenous control (benign prostate tissue). For evaluation of the metastatic cell lines, the “control” (dCT_2_) was considered to be the pT2 tumors. The final result was obtained by applying the 2^–ΔΔct^ method. Findings greater or lesser than 1 were considered to indicate overexpression or underexpression, respectively. All values were standardized relative to the normal control values, which were represented as a value of 1.

### Gene and miRNA selection

The choice of miRNAs and genes evaluated in this study was based on their role in the EMT process in various types of cancer. We performed a literature search via PubMed and Web of Science using the terms “epithelial-mesenchymal transition”, “cancer”, and “miRNA”. Based on the data published in the literature, we selected 18 miRNAs that targeted the most important genes involved in EMT. The data are presented in Table 1.

**Table 1:** Selection of miRNAs and their main targets.

microRNA	Target gene	Reference
**miR-200a**	ZEB1, ZEB2	Bracken *et al*, 2008; Gregory *et al*, 2008; Korpal *et al*, 2008
**miR-200b**	ZEB1, ZEB2, SNAI2, PDGFD	Bracken *et al*, 2008; Gregory *et al*, 2008; Korpal *et al*, 2008; Kong *et al*, 2009; Liu *et al*, 2012
**miR-200c**	ZEB1, ZEB2	Bracken *et al*, 2008; Gregory *et al*, 2008; Korpal *et al*, 2008
**miR-429**	ZEB1, ZEB2	Bracken *et al*, 2008; Gregory *et al*, 2008; Korpal *et al*, 2008
**miR-141**	ZEB1, ZEB2	Burk *et al*, 2008; Gregory *et al*, 2008; Korpal *et al*, 2008
**miR-205**	ZEB1, ZEB2	Gregory *et al*, 2008
**miR-203**	ZEB1, ZEB2, SNAI2	Wellner *et al*, 2009; Saini *et al*, 2011; Zhang *et al*, 2011; Qu *et al*, 2013
**miR-183**	ZEB1	Wellner *et al*, 2009
**miR-1**	SNAI2	Liu *et al*, 2012; Tominaga *et al*, 2012
**miR-29b**	SNAI1	Ru *et al*, 2012
**miR-9**	E-cadherin	Ma *et al*, 2010
**miR-21**	SNAI1	Bornachea *et al*, 2012
**miR-495**	E-cadherin	Hwang-Verslues *et al*, 2011
**miR-30a**	SNAI1, Vimentin	Kumarswamy *et al*, 2011; Cheng *et al*, 2012;
**miR-34a**	ZEB1, SNAI1	Siemens *et al*, 2011; Hahn *et al*, 2013
**miR-155**	TGFB1	Kong *et al*, 2008; Johansson *et al*, 2013
**miR-10b**	E-cadherin, TWIST1	Ma *et al*, 2007; Liu *et al*, 2012
**miR-373**	Involved in metastasis	Huang *et al*, 2008; Yang *et al*, 2009

### Statistical Analysis

To compare the clinicopathological features among patients with localized PCa, the patients were divided into groups based on their GS (GS ≤6 vs GS ≥8), pathological stage (pT2 vs pT3), pre-operative PSA (<10 vs ≥10 ng/mL), and absence or presence of biochemical recurrence, defined as PSA ≥0,02 ng/mL. The patients were also classified into low-risk and high-risk disease groups according to the presence of any unfavorable feature. In this scenario, the expression values in the tumor tissue were compared to those in the benign prostate tissue.

For the evaluation of metastatic tumors, three metastatic PCa cell lines were analyzed together and designated as the metastatic group. The expression levels of the genes and miRNAs between the cell group and pT3 tumors were compared in relation to pT2 tumors, which were considered the “control” group. The rationale was that the pathological stage might represent a practical evidence of EMT, and by using this method, we could evaluate which EMT markers are involved in the progression of a localized tumor to metastasis.

The Mann-Whitney U and T tests were used to compare the GS, pathological stage, pre-operative PSA levels, biochemical recurrence, and risk groups. The distribution of gene and miRNA expression levels was skewed, and the data were log-transformed for analysis. Kaplan-Meier curves were constructed to analyze biochemical recurrence-free survival. The statistical significance for all tests, as assessed by calculating two-sided *P*-values, was set at <0.05.

## Results

### Patient data

The mean age of the patients was 65 years. The mean and median GS were 7.3 and 7, respectively. Twenty-two patients (43%) were stage pT2, and 29 (57%) patients were stage pT3. Seventeen (33%) patients had biochemical recurrence in a mean follow-up period of 63.06 months. The data are illustrated in Table 2.

**Table 2:** Clinicopathological Features of 51 Patients with Localized Prostate Cancer Treated by Radical Prostatectomy.

Clinicopathological Features	PCa Cases (51)	Control (10)	*P*
**Age, years**			
Mean (SD)	65 (±7.5)	71,9 (±8.4)	
Median	66	72	
Min-Max	49–77	59–88	
			0.012
**Clinical Stage (N, %)**			
T1c	22 (45)		
T2a	13 (27)		
T2b	9 (18)		
T2c	5 (10)		
**PSA, ng/dL**			
Mean	8.19 (4.3)	1.05 (0.5)	
Median	9	1.25	
Min-Max	4.1–20	0.06–1.58	
			<0.001
<10 (N, %)	39 (76)		
≥10 (N, %)	12 (24)		
**Gleason Score (N, %)**			
Median GS	7		
Score ≤6	15 (30)		
Score 5	2		
Score 6	13		
Score 7	13 (25)		
Score ≥8	23 (45)		
Score 8	18		
Score 9	3		
Score 10	2		
**Pathologic T Stage (N, %)**			
pT2	22 (43)		
pT3	29 (57)		
**Tumor recurrence (N, %)**			
Yes	17 (33)		
No	34 (67)		

### miRNA and gene expression profiling in localized PCa

miRNAs 200a, 200b, 200c, 429, 141, 205, 203, 21, 183, and 373 were overexpressed in 35 (69%), 47 (92%), 38 (74%), 39 (77%), 42 (82%), 44 (86%), 38 (74%), 51 (100%), 38 (74%), and 33 (64%) samples, respectively. miRNAs 1, 29b, 9, and 495 were underexpressed in 41 (80%), 41 (80%), 36 (71%), and 42 (82%) samples, respectively. miRNAs 34a, 155, 30a, and 10b showed a variable pattern of expression: miR-34a and miR-155 were underexpressed in 55% and 57% of the samples, respectively, and miR-30a and miR-10b were overexpressed in 51% of the samples (Table S1 in File S1).

E-cadherin was overexpressed in 50 cases (98%). The genes N-cadherin, *TGFB1*, and *ZEB1* were underexpressed in 36 (71%) patients, while *SNAI2* and Vimentin were underexpressed in 42 (82%) and 41 (80%) patients, respectively. *ZEB2*, *SNAI1*, and *PDGFD* showed variable patterns of expression. On the other hand, *TWIST1* was the only EMT-induced gene that showed overexpression in the majority of cases (73%) (Table S1 in File S1).

### miRNAs and genes associated with clinicopathological features

Tables 3 and 4 illustrate the data regarding miRNA and gene expression in relation to clinicopathological features, respectively. Low levels of miR-200b, miR-30a, and miR-1 were associated with pT3 disease. Of the 18 miRNAs studied, three were significantly underexpressed in pT3 disease (miR-200b - 7.73 vs 23.86, *P* = 0.02; miR-30a - 1.73 vs 3.79, *P* = 0.048; and miR-1 - 0.72 vs 1.97, *P* = 0.04). However, regarding the genes, we could not find any association between their expression and pathological stage.

**Table 3:** Mean Expression Values and Standard Deviations of miRNAs in Relation to Clinicopathological Features.

	Pathological Stage			Gleason Score			Pre-operative PSA			Biochemical Recurrence			Risk Group		
	Mean			Mean			Mean			Mean			Mean		
	(SD)			(SD)			(SD)			(SD)			(SD)		
miRNA	pT2	pT3	*P*	≤6	≥8	*P*	<10	≥10	*P*	No	Yes	*P*	Low	High	*P*
**200a**	4.03	11.22	0.388	2.41	11.77	0.401	4.61	18.67	0.938	10.31	3.74	0.453	2.72	9.43	0.519
	(6.51)	(38.20)		(2.47)	(42.35)		(8.17)	(56.29)		(35.50)	(4.38)		(2.82)	(32.39)	
**200b**	23.86	7.74	**0.02**	18.67	6.94	**0.035**	16.85	8.98	0.412	18.65	6.78	0.139	21.44	13.04	0.422
	(42.40)	(8.47)		(18.05)	(6.36)		(33.99)	(8.03)		(35.16)	(6.19)		(19.54)	(31.23)	
**200c**	4.10	3.35	0.636	3.52	3.17	0.821	3.59	4.18	0.743	4.06	2.90	0.483	3.18	3.79	0.757
	(6.31)	(4.91)		(3.88)	(5.05)		(5.41)	(6.10)		(6.29)	(3.49)		(3.34)	(5.94)	
**429**	9.45	6.46	0.505	5.99	8.10	0.625	8.68	5.29	0.436	8.57	6.10	0.093	7.74	7.75	0.998
	(17.82)	(14.02)		(6.59)	(15.61)		(18.16)	(4.34)		(19.03)	(4.11)		(7.34)	(17.18)	
**141**	21.98	14.85	0.440	18.64	13.88	0.416	20.23	11.82	0.321	17.74	18.28	0.956	13.53	18.99	0.635
	(44.95)	(17.85)		(17.32)	(17.57)		(36.79)	(12.05)		(37.07)	(20.38)		(13.96)	(35.35)	
**205**	31.09	8.39	0.159	29.41	10.75	0.555	19.85	13.78	0.561	16.63	21.30	0.665	39.61	12.96	0.200
	(50.98)	(10.19)		(51.68)	(12.15)		(40.96)	(15.85)		(30.50)	(45.39)		(61.38)	(24.64)	
**203**	11.47	2.79	0.193	2.40	3.23	0.405	7.72	3.42	0.587	8.28	3.05	0.491	2.04	7.63	0.535
	(38.21)	(3.13)		(2.48)	(3.25)		(29.63)	(2.68)		(30.83)	(3.66)		(1.62)	(28.11)	
**183**	10.53	7.70	0.479	7.79	7.72	0.989	9.03	8.64	0.958	11.21	4.33	0.299	10.13	8.63	0.763
	(15.60)	(12.72)		(11.37)	(14.20)		(13.86)	(15.15)		(16.22)	(5.74)		(13.21)	(14.28)	
**21**	108.96	67.34	0.432	60.48	105.61	0.417	85.29	85.30	0.950	96.75	62.38	0.537	68.24	89.46	0.749
	(213.46)	(161.82)		(106.85)	(193.84)		(169.55)	(237.02)		(214.95)	(104.17)		(126.44)	(197.75)	
**373**	0.29	0.28	0.231	0.34	0.28	0.108	0.28	0.30	0.927	6.16	2.79	0.169	0.26	0.29	0.186
	(0.37)	(0.31)		(0.39)	(0.28)		(0.34)	(0.31)		(8.29)	(4.32)		(0.37)	(0.32)	
**1**	1.97	0.72	**0.040**	2.47	0.68	0.101	1.42	0.82	0.504	1.52	0.73	0.119	2.59	0.93	0.112
	(3.32)	(1.39)		(3.91)	(1.48)		(2.70)	(1.79)		(2.79)	(1.59)		(4.43)	(1.63)	
**29b**	0.74	0.42	0.123	0.76	0.31	0.224	0.51	0.68	0.546	0.57	0.53	0.780	0.51	0.56	0.852
	(0.77)	(0.70)		(0.96)	(0.32)		(0.66)	(0.98)		(0.72)	(0.81)		(0.64)	(0.77)	
**9**	1.44	1.13	0.622	0.83	1.75	0.930	0.92	2.17	0.415	0.89	2.01	0.281	0.96	1.34	0.637
	(2.86)	(1.69)		(0.84)	(3.03)		(1.39)	(1.59)		(1.27)	(3.41)		(0.89)	(2.47)	
**495**	1.03	0.81	0.623	0.66	0.92	0.536	0.81	1.13	0.479	0.95	0.80	0.752	0.77	0.93	0.780
	(0.35)	(0.33)		(0.41)	(0.33)		(0.37)	(0.31)		(1.73)	(1.18)		(0.39)	(0.32)	
**34a**	3.81	4.92	0.690	2.79	5.44	0.433	4.53	4.20	0.976	4.03	5.25	0.675	1.02	5.27	0.924
	(7.93)	(10.92)		(7.69)	(11.31)		(10.31)	(8.35)		(8.11)	(12.47)		(0.58)	(10.64)	
**155**	2.83	1.89	0.405	3.90	1.65	0.260	1.97	3.16	0.281	2.40	2.08	0.788	3.12	2.09	0.463
	(4.22)	(3.73)		(5.84)	(2.95)		(3.41)	(5.28)		(3.69)	(4.49)		(4.56)	(3.80)	
**30a**	3.79	1.73	**0.048**	4.73	2.13	0.425	2.72	2.34	0.877	3.43	0.99	0.145	6.37	1.70	**0.039**
	(5.85)	(3.14)		(6.92)	(3.32)		(4.72)	(4.46)		(5.39)	(1.13)		(7.91)	(2.77)	
**10b**	5.22	4.32	0.760	8.75	2.69	0.680	4.74	4.63	0.967	5.52	3.10	0.434	3.95	4.89	0.798
	(8.13)	(11.79)		(16.51)	(6.14)		(10.75)	(9.67)		(11.22)	(8.18)		(7.16)	(10.98)	

**Table 4:** Mean Expression Values and Standard Deviations of Genes in Relation to Clinicopathological Features.

	Pathological Stage			Gleason Score			Pre-operative PSA			Biochemical Recurrence			Risk Group		
	Mean			Mean			Mean			Mean			Mean		
	(SD)			(SD)			(SD)			(SD)			(SD)		
Gene	pT2	pT3	*P*	≤6	≥8	*P*	<10	≥10	*P*	No	Yes	*P*	Low	High	*P*
**E-cadherin**	4.61	6.10	0.343	5.07	5.75	0.736	5.74	4.72	0.644	5.70	4.98	0.667	3.06	6.04	0.126
	(3.39)	(6.68)		(8.19)	(4.12)		(6.14)	(3.45)		(6.29)	(3.60)		(1.61)	(5.96)	
**TGFB1**	0.68	1.04	0.123	0.77	1.06	0.497	0.89	0.87	0.994	0.83	1.01	0.335	0.69	0.94	0.263
	(0.31)	(0.75)		(0.43)	(0.81)		(0.55)	(0.83)		(0.59)	(0.69)		(0.38)	(0.66)	
**ZEB1**	1.15	1.10	0.903	1.03	1.13	0.803	1.21	0.90	0.520	1.19	0.98	0.580	1.13	1.12	0.980
	(1.58)	(1.02)		(1.02)	(1.14)		(1.34)	(1.15)		(1.44)	(0.88)		(1.23)	(1.31)	
**ZEB2**	1.23	1.45	0.459	1.48	1.38	0.764	1.53	0.89	0.50	1.41	1.24	0.577	1.32	1.36	0.904
	(1.01)	(1.02)		(0.99)	(1.08)		(1.12)	(0.44)		(1.07)	(0.91)		(1.08)	(1.01)	
**TWIST1**	6.51	8.25	0.665	3.62	4.78	0.543	4.76	14.75	0.445	9.48	3.53	0.153	1.81	8.89	**0.018**
	(10.14)	(16.43)		(5.54)	(5.78)		(5.33)	(25.16)		(16.69)	(3.14)		(2.34)	(15.25)	
**SNAI1**	2.80	2.44	0.728	1.47	2.66	0.637	2.73	2.24	0.761	3.11	1.58	0.072	1.21	2.94	0.602
	(4.07)	(3.25)		(1.65)	(3.64)		(3.90)	(2.76)		(4.06)	(2.15)		(0.85)	(3.92)	
**N-cadherin**	0.86	1.61	0.288	1.74	1.19	0.561	1.39	1.01	0.641	1.44	0.97	0.525	0.81	1.40	0.496
	(0.96)	(3.13)		(4.22)	(1.25)		(2.81)	(1.16)		(2.94)	(0.88)		(0.91)	(2.69)	
**Vimentin**	0.67	2.80	0.533	0.55	0.63	0.760	0.76	0.83	0.692	0.88	0.58	0.315	0.27	0.90	**0.017**
	(0.75)	(1.17)		(0.96)	(0.79)		(0.99)	(1.08)		(1.06)	(0.89)		(0.28)	(1.08)	
**SNAI2**	0.76	0.93	0.684	0.75	0.48	0.422	1.01	0.46	0.382	1.08	0.39	0.369	0.29	0.99	0.176
	(1.33)	(1.58)		(1.50)	(0.47)		(1.68)	(0.43)		(1.74)	(0.27)		(2.34)	(1.60)	
**PDGFD**	1.91	1.30	0.985	1.06	1.39	0.423	1.68	1.25	0.596	1.85	0.98	0.562	1.21	1.65	0.582
	(3.09)	(1.21)		(0.97)	(1.37)		(2.49)	(1.31)		(2.65)	(0.60)		(1.13)	(2.42)	

We assessed the association of GS with the miRNAs excluding GS 7 because of their uncertain behavior. Fifteen patients (29%) had a GS ≤6 and 23 (45%) had a GS ≥8. We found that miR-200b expression was significantly lower in patients with a GS ≥8 when compared to patients with a GS ≤6 (6.94 vs 18.67, *P* = 0.035). No association was found between GS and the other miRNAs and genes.

When patients were grouped according to low-risk and high-risk disease, the high-risk disease had significantly lower levels of miR-30a (1.70 vs 6.37, *P* = 0.039). Also high levels of Vimentin and *TWIST1* were significantly associated with high-risk disease (0.27 vs 0.90, *P* = 0.017; 1.81 vs 8.89, *P* = 0.018).

**Figure 1:**
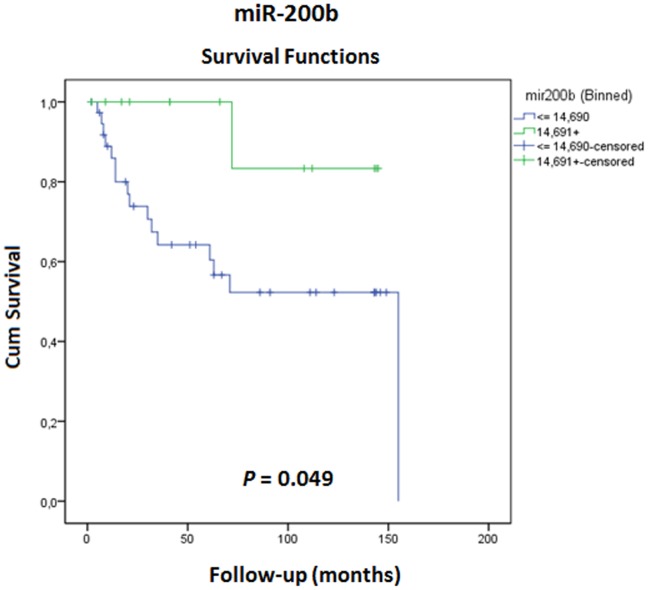
Kaplan-Meier biochemical recurrence-free survival curve based on miR-200b mean expression (P = 0.049, Log rank test). Patients with miR-200b expression levels ≤14.690 showed significantly shorter biochemical recurrence-free survival.

Due to the significant association between miRNAs 200b, 30a, and 1 with pathological stage and their potential as prognostic markers, a survival analysis was performed. Kaplan-Meier analysis revealed that patients with lower levels of miR-200b had significantly shorter biochemical recurrence-free survival (*P* = 0.049) (Figure 1).

Moreover, miR-183 and *TWIST1* expression levels were significantly higher in metastatic PCa cell lines compared to the levels in patients with pT3 disease and high-grade tumors (Table S2 in File S1). In cell lines, the miR-183 and Twist1 levels were 2.64 and 3.54, respectively, while in pT3 tumors, their levels were 40.41 and 14.45, respectively (*P* = 0.009 and *P* = 0.049, respectively).

## Discussion

**Figure 2:**
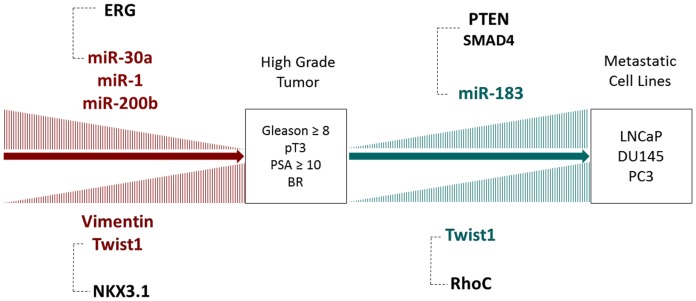
Main miRNAs and genes involved in epithelial-mesenchymal transition in prostate cancer. Expression levels of miRNAs 200b, 30a and 1 decrease when the tumor acquires high grade features, while expression levels of TWIST1 and Vimentin increase. When the tumor becomes metastatic, an increase in the expression levels of miR-183 and TWIST1 is observed. The dotted lines indicate the genes where these miRNAs or genes might act, based on previously published data. *BR*  =  Biochemical Recurrence.

The importance of the EMT in carcinogenesis has been extensively studied in the last few years, and it is now considered one of the main mechanisms responsible for tumor progression and metastatic dissemination. Our study aimed to evaluate the significance of the expression patterns of multiple miRNAs and genes involved in EMT in clinical specimens of localized prostate cancer and in metastatic cell lines. Our findings are summarized in Figure 2, which shows the main miRNAs and genes involved in EMT in the progression of PCa and their possible mechanism of action.

We have shown that miR-200b, miR-30a, and miR-1 were significantly underexpressed in non-organ-confined tumors and could constitute interesting prognostic factors. A recent study supports our findings by showing that miR-200b and miR-1 induce mesenchymal-epithelial transition (MET) in mouse and human PCa cells and are important regulators in prostatic tumorigenesis and tumor progression [23].

miR-200b was overexpressed in PCa specimens, and this finding is in agreement with previous studies on PCa [25], [26]. The members of the miR-200 family are the most important miRNAs involved in EMT [27], and studies in PCa cells have shown that miR-200b inhibits EMT, growth, and metastasis [23], [28]. We hypothesize that miR-200b has the greatest potential to become a prognostic marker because lower expression of miR-200b was significantly associated with a high GS, pT3 disease, and shorter biochemical recurrence-free survival. The role of miR-200b has been described in other tumors, and its downregulation is related to advanced disease stage [29] and shorter overall survival [30]–[32]. Similar to our findings, Barron et al found that miR-200a levels were reduced in patients who relapsed by studying miR-200a expression in formalin-fixed paraffin-embedded tissue from patients with pT3 disease [33], supporting the potential of miR-200 family as a marker of biochemical recurrence.

Previous studies indicate that downregulation of miR-200 may contribute to the progression of PCa [15] [34]. Xu et al observed an 80% reduction in miR-200b levels in chemically castrated LNCaP cells via RNA sequencing [35]. Emerging evidence supports the involvement of EMT processes in the deregulation of the androgen signaling axis, but data are still controversial. Zhu and Kyprianou observed that androgens induce independently EMT patterning within prostate cancer cells, resulting in substantial changes in cellular invasion and motility [36]. The activated androgen receptor (AR) has recently been shown to promote EMT activation via suppression of E-cadherin expression within breast cancer cells [37]. On the other hand, Sun et al found that androgen deprivation causes EMT in vivo and acquisition of mesenchymal features [38].

This is the second study relating miR-1 and prognosis in PCa. Hudson et al previously found that lower expression levels of miR-1 were associated with earlier biochemical recurrence in PCa [39]. Now we showed that miR-1 was downregulated in the primary tumor compared to benign prostate tissue, and was significantly reduced in non-organ confined disease. It is thought that miR-1 regulating Slug [23], through histone methylation and acetylation [39] and also having as target genes related to proliferation, migration and invasion [40] plays an important role in EMT in PCa.

Data regarding the role of miR-30a in PCa are scarce and contradictory. In our study, miR-30a showed a variable pattern of expression. miR-30a was described as being downregulated in the study conducted by Porkka et al [41], while Carlsson et al reported the upregulation of this miRNA [42]. Recently, Kao et al showed that the ETS-related gene (*ERG*), which is the most frequently overexpressed oncogene in PCa, is a direct target of miR-30 and that overexpression of miR-30 in PCa cells suppresses EMT phenotypes and inhibits cell migration and invasion [43]. miR-30 family also inhibits cell migration, invasiveness, and metastasis *in vitro* in other tumors, such as lung, breast, and hepatocellular cancer [21], [44]–[46], by targeting *SNAI1* [21], [44] and Vimentin [45], [46]. In this study, the relationship observed between decreased expression of miR-30, advanced pathological stage, and high-risk disease confirms miR-30 as a tumor suppressor miRNA in PCa. Cheng et al observed that low levels of miR-30a were predictors of advanced stage and lymph node metastasis in invasive breast cancer [45]. Wang et al showed that low expression levels of miR-30a were significantly associated with a higher incidence of portal vein tumor thrombus in hepatocellular carcinoma [46].

Regarding the genes, we observed overexpression of E-cadherin in virtually all cases, and the majority of the mesenchymal markers, including N-cadherin, *TGFB1*, *ZEB1*, Vimentin, and *SNAI2*, were downregulated. This gene expression profile strongly suggests that localized PCa maintains the epithelial phenotype despite tumor differentiation and increasing stage. However, *TWIST1* was overexpressed in 73% of the cases. *TWIST1* is a helix-loop-helix transcription factor that activates EMT through indirect inhibition of E-cadherin [47]. *TWIST1* has been shown to be overexpressed in PCa on immunohistochemistry assays and to positively correlate with the GS [48], [49]. It is interesting that a gene with such importance in EMT and with prognostic value in PCa is overexpressed in localized tumors. The early overexpression of *TWIST1* may be attributed to its regulation by the *NKX3-1* gene [50], a tumor suppressor that was found to be underexpressed in the early stages of PCa [51], [52]. However, the early upregulation of *TWIST1* does not appear to be sufficient to initiate the EMT process. According to Casas et al, *TWIST1* induces *SNAI2* to promote EMT [53], but depletion of *SNAI2* completely blocks the ability of *TWIST1* to suppress E-cadherin and induce EMT.

We have shown that high levels of *TWIST1*, as well as Vimentin, are significantly associated with patients in the high-risk group and *TWIST1* were also significantly higher in the metastatic cell lines. In a recent study, Behnsawy et al [12] showed that high expression levels of *TWIST1* and Vimentin evaluated by immunohistochemistry is an independent factor related to shorter biochemical recurrence-free survival, suggesting that these genes might be potential markers of biochemical recurrence after radical prostatectomy.

*TWIST1* appears to play a role in various steps of EMT, and its role in the progression of PCa [49]. In the study by Kwok et al, *TWIST1* expression was higher in tissues derived from metastatic lesions from bones and lymph nodes [48]. The role of *TWIST1* in this later step of EMT might be explained by the activation of its target, miR-10b. miR-10b not only represses E-cadherin [54] but also inhibits the translation of the HOXD10 protein, permitting the expression of the pro-metastatic gene product, *RHOC* [55].

We have also observed that expression levels of miR-183 were significantly higher in the metastatic group. Ueno et al observed that higher expression levels of miR-183 were significantly associated with higher PSA, higher stage and shorter overall survival after radical prostatectomy, but its behavior in PCa is absolutely controversial some showing that miR-183 promotes migration and invasion [56]–[58], while others indicate that it inhibits migration, invasion, and metastasis [59]–[61]. Some targets of miR-183 have been proposed, *DKK3*, *SMAD4* [56], *EGR1* and *PTEN* [57], which turns miR-183 a context dependent miRNA. Based on our results and according to previous studies in the literature, we believe that miR-183 acts as an oncomiR in PCa, and the mechanism might involve *PTEN* which is related to PCa progression and development of metastasis [62]. Ding et al also showed that concomitant *PTEN* and *SMAD4* inactivation in the prostatic epithelium is able to produce a fully-penetrant invasive and metastatic PCa phenotype in mice [63].

In conclusion, it is important to understand that EMT influences tumor progression in different steps through several markers. Here, we described a comprehensive study of miRNAs and genes related to EMT in PCa and found that the expression levels of miR-200b, miR-30a, miR-1, *TWIST1* and Vimentin could be used in decision-making processes related to primary or adjuvant treatments in the future.

## Supporting Information

File S1 **Combined file of supporting tables.** Table S1: Expression levels of miRNAs and genes from each case in relation to BPH samples. Table S2: Expression levels of miRNAs and genes in pT3 tumors and in cell lines (in relation to pT2 tumors)
(DOCX)
