# Supplementary material for: Comprehensive Study of Gene and microRNA Expression Related to Epithelial-Mesenchymal Transition in Prostate Cancer
Source: PLoS One. 2014 Nov 19;9(11):e113700. doi: 10.1371/journal.pone.0113700 (PMC4237496; doi:10.1371/journal.pone.0113700)
Supplement: File S1 — Combined file of supporting tables. Table S1: Expression levels of miRNAs and genes from each case in relation to BPH samples. Table S2: Expression levels of miRNAs and genes in pT3 tumors and in cell lines (in relation to pT2 tumors) (DOCX) [file pone.0113700.s001.docx]

Table S1. Expression levels of miRNAs and genes from each case in relation to BPH samples

Table S2. Expression levels of miRNAs and genes in pT3 tumors and in cell lines (in relation to pT2 tumors)

Mean Expression Values and Standard Deviations of miRNAs and Genes in pT3 Tumors and in Metastatic Cell Lines in Relation to pT2 Tumors

|  | Metastatic PCa cell lines  Mean  (SD) | | |  | Metastatic PCa cell lines  Mean  (SD) | | |  | Metastatic PCa cell lines  Mean  (SD) | | |
| --- | --- | --- | --- | --- | --- | --- | --- | --- | --- | --- | --- |
| miRNA | **pT3** | **Cell** | ***P*** | miRNA | **pT3** | **Cell** | ***P*** | **Gene** | **pT3** | **Cell** | ***P*** |
| 200a | 8.18  (27.83) | 0.51  (0.73) | 0.642 | **1** | 1.09  (2.09) | 0.0007  (0.001) | 0.382 | **E-cadherin** | 1.63  (1.79) | 0.81  (1.10) | 0.446 |
| 200b | 0.77  (0.84) | 1.31  (0.96) | 0.310 | **29b** | 1.11  (1.87) | 6.21  (9.58) | 0.317 | **TGFB1** | 1.76  (1.27) | 3.98  (3.47) | 0.383 |
| 200c | 1.73  (2.53) | 0.06  (0.10) | 0.271 | **9** | 1.98  (2.97) | 37.10  (59.33) | 0.258 | **ZEB1** | 1.52  (1.41) | 0.27  (0.31) | 0.140 |
| 429 | 1.83  (3.98) | 0.65  (0.65) | 0.615 | **495** | 1.96  (3.05) | 0.02  (0.008) | 0.287 | **ZEB2** | 1.49  (1.02) | 0.03  (0.02) | 0.057 |
| 141 | 3.43  (6.71) | 3.50  (3.31) | 0.985 | **34a** | 4.72  (10.50) | 0.81  (0.57) | 0.530 | **TWIST1** | 3.54  (7.05) | 14.44  (11.37) | **0.049** |
| 205 | 0.96  (1.16) | 0.001  (0.002) | 0.171 | **155** | 1.52  (3.01) | 0.02  (0.04) | 0.400 | **SNAI1** | 2.05  (2.72) | 3.09  (2.78) | 0.533 |
| 203 | 0.98  (1.10) | 2.50  (4.22) | 0.497 | **30a** | 1.43  (2.59) | 0.73  (0.55) | 0.651 | **N-cadherin** | 3.05  (5.94) | 0.99  - | 0.737 |
| 183 | 2.64  (4.36) | 40.41  (51.87) | **0.009** | **10b** | 2.43  (6.64) | 0.07  (0.06) | 0.548 | **Vimentin** | 2.48  (3.38) | 4.78  (3.87) | 0.275 |
| 21 | 2.79  (6.71) | 8.57  (8.96) | 0.177 |  |  |  |  | **SNAI2** | 2.83  (4.81) | 2.11  (1.51) | 0.799 |
| 373 | 1.11  (1.47) | 1.08  (1.03) | 0.975 |  |  |  |  | **PDGFD** | 1.36  (1.27) | 0.53  (0.48) | 0.273 |
